# Change in incidence rates for psychosis in different ethnic groups in south London: findings from the Clinical Record Interactive Search-First Episode Psychosis (CRIS-FEP) study

**DOI:** 10.1017/S0033291719003234

**Published:** 2021-01

**Authors:** Sherifat Oduola, Jayati Das-Munshi, Francois Bourque, Charlotte Gayer-Anderson, Jason Tsang, Robin M. Murray, Tom K. J Craig, Craig Morgan

**Affiliations:** 1 School of Health Sciences, University of East Anglia, Norwich Research Park, Norwich NR4 7TJ, UK; 2 Department of Health Service and Population Research, Institute of Psychiatry, Psychology & Neuroscience, King's College London, De Crespigny Park, Denmark Hill, London SE5 8AF, UK; 3 South London & Maudsley NHS Foundation Trust, Denmark Hill, London SE5 8AZ, UK; 4 Department of Psychological Medicine, Institute of Psychiatry, Psychology & Neuroscience, King's College London, De Crespigny Park, Denmark Hill, London SE5 8AF, UK; 5 Division of Social and Cultural Psychiatry, Douglas Mental Health University Institute, McGill University, Montreal (Quebec), H4H 1R3, Canada; 6 Department of Psychosis Studies, Institute of Psychiatry, Psychology & Neuroscience, King's College London, De Crespigny Park, Denmark Hill, London SE5 8AF, UK

**Keywords:** Black African, black Caribbean, ethnic minority, ethnicity, migrant, psychosis

## Abstract

**Background:**

A higher incidence of psychotic disorders has been consistently reported among black and other minority ethnic groups, particularly in northern Europe. It is unclear whether these rates have changed over time.

**Methods:**

We identified all individuals with a first episode psychosis who presented to adult mental health services between 1 May 2010 and 30 April 2012 and who were resident in London boroughs of Lambeth and Southwark. We estimated age-and-gender standardised incidence rates overall and by ethnic group, then compared our findings to those reported in the Aetiology and Ethnicity of Schizophrenia and Other Psychoses (ÆSOP) study that we carried out in the same catchment area around 10 years earlier.

**Results:**

From 9109 clinical records we identified 558 patients with first episode psychosis. Compared with ÆSOP, the overall incidence rates of psychotic disorder in southeast London have increased from 49.4 (95% confidence interval (CI) 43.6–55.3) to 63.1 (95% CI 57.3–69.0) per 100 000 person-years at risk. However, the overall incidence rate ratios (IRR) were reduced in some ethnic groups: for example, IRR (95% CI) for the black Caribbean group reduced from 6.7 (5.4–8.3) to 2.8 (2.1–3.6) and the ‘mixed’ group from 2.7 (1.8–4.2) to 1.4 (0.9–2.1). In the black African group, there was a negligible difference from 4.1 (3.2–5.3) to 3.5 (2.8–4.5).

**Conclusions:**

We found that incidence rates of psychosis have increased over time, and the IRR varied by the ethnic group. Future studies are needed to investigate more changes over time and determinants of change.

## Introduction

A higher incidence of psychotic disorders has been consistently reported among black and some other minority ethnic groups, particularly in northern European countries (Fearon *et al*., 2006; Coid *et al*., 2008; Veling, 2013). These rates are generally higher compared with rates in the countries of origin or heritage (Jablensky *et al*., 1992*b*; Hickling and Rodgers-Johnson, 1995; Bhugra *et al*., 2000; Selten *et al*., 2001; Selten *et al*., 2002). This suggests that social and environmental factors in host countries underpin the high rates. In addition, incidence rates are not similarly elevated across all minority ethnic groups. For example, in the UK the highest reported rates are for black ethnic groups, with incidence rate ratios (IRR) ranging from 2.1 to 18.2 for black Caribbean (Lloyd *et al*., 2005; Cheng *et al*., 2011) and from 2.5 to 11.9 for black African (Lloyd *et al*., 2005; Kirkbride *et al*., 2008). In the Netherlands, reported relative risks (RRs) of psychosis range from 2.3 to 7.8 for Moroccan populations (Veling *et al*., 2006; Zandi *et al*., 2010) and 1.4 to 4.0 for Surinamese populations (Selten *et al*., 2001; Veling *et al*., 2007). It is unclear whether these rates have changed over time.

Early research on rates of psychotic disorders in minority ethnic groups was characterised by considerable methodological heterogeneity. For example, many studies used hospital admissions to identify those with a first episode psychotic disorder, which meant that findings were confounded by other factors, such as mode of contact, since not all individuals with a psychotic disorder are admitted to hospitals (Kendell *et al*., 1993). Such a design may introduce systematic bias that may exacerbate differences between ethnic groups. More recent studies have brought a measure of methodological rigour, such as population-based studies that include both hospital and community patients (Fearon *et al*., 2006; Coid *et al*., 2008; Cheng *et al*., 2011; Kirkbride *et al*., 2017). Further, some previous studies grouped all black patients and therefore do not allow estimates of rates within quite different black Caribbean and black African groups.

While some studies have estimated the incidence of psychosis specifically among black Caribbean populations (Hutchinson *et al*., 1996; Harrison *et al*., 1997), to date, only three epidemiological studies in the UK have investigated incidence rates and rate ratios among black African and black Caribbean groups separately, namely the Aetiology and Ethnicity of Schizophrenia and Other Psychoses (ÆSOP), the East London First Episode Psychosis and the Social Epidemiology of Psychoses in East Anglia studies (Fearon *et al*., 2006; Coid *et al*., 2008; Kirkbride *et al*., 2017). All have reported an increased incidence of psychosis in black African and black Caribbean populations compared with white British populations.

There are of course considerable differences between the different migrant populations. For example, some minority ethnic groups are more integrated into UK society than others. For instance, the migration of black African people to the UK is more recent compared with black Caribbeans, whose migration to the UK was most notable during the 1940s and 1950s (Mason, 1995; Chamberlain, 2002). This is an important marker of population change, which may have an impact on the mental health of the population. Given the limited research investigating rates of psychosis among black African and black Caribbean populations separately, far less is known about whether the elevated rates persist in these two groups. If the elevated incidence is persistent, this further strengthens the urgent need for public health strategies to address these disparities.

ÆSOP was a large prospective incidence study of psychotic disorders among minority ethnic groups between 1997 and 1999 in three catchment areas in England, namely, southeast London, Nottingham and Bristol (Fearon *et al*., 2006). The sample comprised first episode cases aged 16–64 years, the majority (66%) of whom were drawn from the southeast London site. This study found that the incidence of psychosis was higher among minority ethnic groups compared with white British. ÆSOP provides a methodological template to re-examine the evidence on incidence among ethnic minority populations, particularly the black African and black Caribbean groups in southeast London. In this current study, we addressed the question of whether the increased incidence of psychosis among these groups had changed 10 years later.

We therefore set out to compare rates in 2010–2012 in southeast London with those reported in 1997–1999 as part of the ÆSOP study. We used data from one of the largest anonymous electronic health record systems in Europe, covering an ethnically diverse population in the UK to estimate the incidence of psychosis by age, gender and ethnicity over a two year period, modelled on methods used in previous face-to-face epidemiological studies such as ÆSOP.

## Methods

### Study design and population at risk

Using the South London and Maudsley NHS Foundation Trust (SLaM) Biomedical Research Centre (BRC) Clinical Records Interactive Search (CRIS) system (Stewart *et al*., 2009), we sought to identify all individuals with a first episode psychotic disorder (in ICD-10: F20-F29, F30-F33 psychotic codes) who presented to mental health services in the SLaM between 1 May 2010 and 30 April 2012 and who were resident in the London boroughs of Lambeth and Southwark. The first year of this study was conducted as part of the European Union Gene-Environment Interactions study (Jongsma *et al*., 2018).

### Study setting and participants

The study was conducted within two inner city areas in southeast London, UK. The areas covered were the London boroughs of Lambeth (total population, 303 086) and Southwark (total population, 288 283) (ONS, 2011*b*), served by SLaM. These areas have large minority ethnic populations, principally black African (28.0%) and black Caribbean (15.7%) (ONS, 2011*b*). SLaM is one of the largest mental health providers in Europe serving a population over 1.3 million across four south London boroughs (Stewart *et al*., 2009). Since April 2006, full electronic health records have been operational in SLaM. Between 2007 and 2008, the CRIS was built, which provides a fully anonymised copy of SLaM electronic records (Perera *et al*., 2016). CRIS contains over 300 000 patient records from hospital and community services. The clinical information documented in CRIS appears in two forms i.e. structured fields (e.g. dates, demographic and diagnosis) and unstructured free text fields (e.g. clinical information in medical notes and correspondence) (Perera *et al*., 2016). We interrogated the CRIS database for clinical and demographic information to screen all potentially eligible patients for inclusion in the study.

### Case identification

First episode psychosis cases were ascertained through a 3-stage manual screening of CRIS clinical records between May 2010 and April 2012. Firstly, a combination of information from the CRIS structured and free text fields was used to ascertain probable cases of psychosis. We used the structured language query (Netz *et al*., 2001; Tulloch, 2013) to interrogate and extract information in CRIS based on our inclusion criteria i.e. using defined search terms (date, postcode, age, symptoms-psychos*; psychot*, delusion*, voices, hallucinat* and diagnosis). This returned a set of patient records, which were individually screened by a team of researchers using the Screening Schedule for Psychosis (Jablensky *et al*., 1992*a*). Secondly, two primary researchers reviewed all the included cases from the first stage screen to ensure cases met all inclusion criteria. Thirdly, discrepant or ambiguous cases were resolved by consensus with the principal investigator (CM) (Oduola *et al*., 2019).

### Inclusion and exclusion criteria

Inclusion and exclusion criteria were the same as those used in the Aetiology and Ethnicity in Schizophrenia and Other Psychoses study (Fearon *et al*., 2006). Cases were included if they were: resident in the London boroughs of Lambeth or Southwark; aged 18–64 years (inclusive); experiencing psychotic symptoms, during the study periods, as assessed by the Screening Schedule for Psychosis (Jablensky *et al*., 1992*a*). Exclusion criteria were: (a) evidence of psychotic symptoms caused by an organic cause, (b) transient psychotic symptoms resulting from acute intoxication and (c) evidence of the previous contact with services for psychotic symptoms.

### Data collection and instruments

We screened for psychotic symptoms using the Screening Schedule for Psychosis (Jablensky *et al*., 1992*a*). Socio-demographic data were collected using the Medical Research Council Socio-demographic Schedule (MRC-SDS) (Mallett, 1997). The MRC-SDC classifies ethnic groups according to the UK Office for National Statistics (ONS) ethnic categories (ONS, 2011*b*). The Personal and Psychiatric History Schedule (WHO, 1996), adapted for case notes, was used to determine the date of onset and the Operational Criteria Checklist for Psychotic Illness (McGuffin *et al*., 1991) to assess psychotic symptoms.

### Reliability

A number of steps were taken to ensure the reliability of screening and data collection procedures. Firstly, researchers were trained in the inclusion and exclusion criteria and the application of these. Interrater reliability was assessed for case identification, whereby researchers who were blind to each other's ratings, swapped their screened cases and repeated the screening procedure. A *κ* of 0.78, *p* < 0.001 was achieved, indicating substantial agreement.

We verified patients' residence using the Public Health England postcode widget (Public Health England, 2004). We then linked the middle layer super output area (MSOA) data for the catchment area (ONS, 2011*b*) to individual patients to ensure their addresses mapped to the boundaries of the study area. The MSOA is a geographic hierarchy designed for reporting of small area statistics that therefore provides neighbourhood-level information (including postcodes) which fits within the boundaries of a local authority (ONS, 2011*b*) By linking the patients' addresses to the MSOA, we were able to confirm that cases were resident in the study catchment area.

### Data

Ethnicity was self-ascribed and recorded in clinical records. Where this information was missing, ethnicity was ascribed independently by researchers using all available information from the free-text field in clinical records, including country of birth, nationality, language spoken at home, parents' country of birth, geographical region (e.g. Saharan and sub-Saharan Africa) and religious group, as recommended by the Office for National Statistics (ONS, 2011*a*). A high inter-rater reliability was achieved between three researchers, who independently extracted and rated ethnicity information on 89 cases (*κ* score = 0.87, *p* < 0.001), indicating substantial agreement.

We coded ethnicity using the MRC-SDC (Mallett, 1997) according to the 18 categories used in the 2011 census. For analytical purposes, we collapsed the ethnic groups into seven categories to match those used in the ÆSOP study (Fearon *et al*., 2006) as follow: white British, black Caribbean (black Caribbean and other black), black African, Asian (Indian, Pakistani, Bangladeshi, Chinese), white non-British (white Irish, white Gypsy, white Other), other (Arab, Any Other Ethnic group) and mixed (all mixed groups).

### Ethical approval

The CRIS system was approved as an anonymised dataset for secondary analysis by the Oxfordshire Research Ethics Committee (reference 08/H0606/71). Local approval for this study was obtained from the CRIS Oversight Committee at the BRC SLaM (reference: 09-041).

### Statistical analysis

Stata version 12 (StataCorp, 2011) was used to analyse the data. Populations were estimated from the 2011 UK Census and stratified by age (5 year age-band i.e. 18–19; 20–24; 25–29; 30–34; 35–39; 40–44; 45–49; 50–54; 55–59; 60–64), gender and ethnicity. Age-gender-standardised incidence rates of psychotic disorders were calculated by direct standardisation, using the population of England and Wales in 2001, as was used in the ÆSOP study. Crude IRR were calculated and then adjusted for confounders (age and gender) using Poisson regression. Finally, we stratified by age-band and gender and estimated IRR for each ethnic group using white British as the reference group.

## Results

### Sample

Our searches of CRIS retrieved 9109 potentially eligible patients who presented to services during the 2-year study period. Of these, 558 met our inclusion criteria, 8549 screened negative (i.e. 5324 did not have a psychotic disorder, 2956 had previous episodes of psychosis and 359 were either not resident in the study area or were older or younger than the age of inclusion) (see online Supplementary Fig. 1). In our sample, the mean age was 33.3 (s.d. = 10.6) and there was a similar proportion of men (52.3%) and women (47.3%) (Table 1). Compared with the population at risk in Lambeth and Southwark, cases were younger and more were from black Caribbean and black African ethnic groups.

**Table 1.** Demographic characteristics of CRIS-FEP study population and crude and age-gender standardised incidence per 100 000 person-years with 95% CI of all psychoses

	Cases *N* = 558 (%)	Population at risk *N* = 852 920 (%)	χ^2^ test (df)	*p* value	Crude incidence rate	Standardised incidence rate (95% CI)
Gender			4.95 (3)	0.17		
Male	292 (52.3)	425 968 (49.9)			68.5	64.5 (56.0–72.9)
Female	266 (47.7)	426 952 (50.1)			62.3	61.8 (53.7–69.9)
Ethnicity			725.3 (18)	<0.001		
White British	133 (23.8)	351 412 (41.2)			37.8	44.3 (35.1–53.5)
Black African	147 (26.3)	107 670 (12.6)			136.5	125.9 (104–147.1)
Black Caribbean	91 (16.3)	87 788 (10.3)			103.7	100.4 (78.9–121.8)
White non-British	75 (13.4)	160 918 (18.9)			46.6	52.6 (36.5–68.7)
Asian	44 (7.9)	74 432 (8.7)			59.1	54.5 (36.0–73.0)
Mixed	27 (4.8)	45 354 (5.3)			59.5	52.1 (27.7–76.5)
Other	41 (7.3)	25 346 (2.9)			161.8	126.4 (85.6–167.2)

### Incidence rates of psychosis by age and gender

**Fig. 1.**
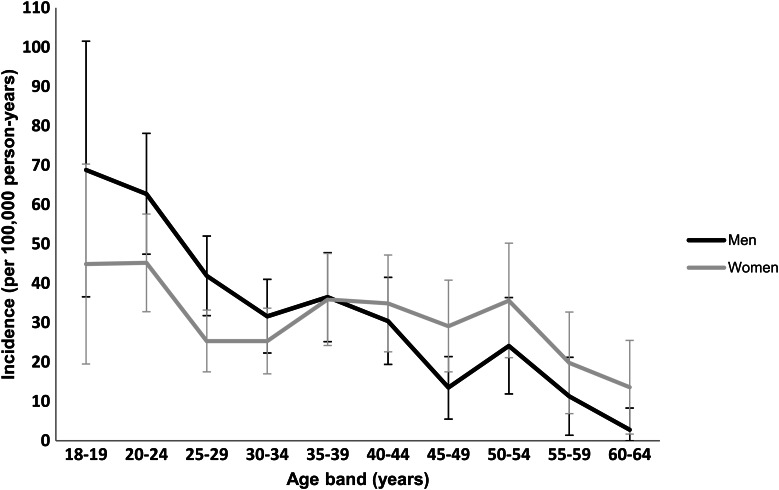
Incidence rates of all psychoses by age and gender: CRIS-FEP study.

The overall age-gender-standardised incidence rate of psychotic disorders was 63.1 per 100 000 person-years at risk (PY) (95% confidence interval (CI) 57.3–69.0). There was no difference in incidence rates between men (64.5 per 100 000 PY) and women (61.8 per 100 000 PY). Figure 1 shows incidence rates by age and gender. Rates were higher among men up to age 35–39 years, while women had higher rates than men from age 40 years onward.

### Incidence rates and rate ratios by ethnic group

The overall age-and-gender standardised incidence rate of psychotic disorders reported in the ÆSOP southeast London site was 49.4 per 100 000 (95% CI, 43.6–55.3) (Kirkbride *et al*., 2006), which is modestly lower than the present CRIS-FEP study. Table 2 shows the comparisons between ÆSOP and the present study in the incidence rate and adjusted rate ratios of psychotic disorders by the ethnic group. In the ÆSOP study, higher incidence rates were reported among all minority ethnic groups compared with the white British group (20.2 per 100 000). These were most markedly raised for black Caribbean (140.8 per 100 000; adj. IRR 6.7; 95% CI 5.4–8.3), black African (80.6 per 100 000; adj. IRR 4.1; 3.2–5.3) and ‘other’ (55.0 per 100 000; adj. IRR 2.6 95% CI 1.7–3.9) groups. Rates were modestly raised for the Asian (31.6 per 100 000; adj. IRR 1.5 95% CI 0.9–2.4), white non-British (33.1 per 100 000; adj. IRR 1.6; 95% CI 1.1–2.2) and ‘mixed’ (45.9 per 100 000; adj. IRR 2.7; 95% CI 1.8–4.2) groups.

**Table 2.** Comparisons between ÆSOP and CRIS-FEP for age-gender standardised incidence per 100 000 person-years with adjusted IRR for all psychoses

	Standardised IR (95% CI) ÆSOP (*Fearon et al., 2006*)	Standardised IR (95% CI) CRIS-FEP study	Adjusted IRR (95% CI) ÆSOP^a^	Adjusted IRR (95% CI) CRIS-FEP study
White British	20.2 (17.8–22.7)	44.3 (35.1–53.5)	1.00	1.00
Black African	80.6 (60.0–101.2)	125.9 (104–147.1)	4.1 (3.2–5.3)	3.5 (2.8–4.5)**
Black Caribbean	140.8 (114.4–167.2)	100.4 (78.9–121.8)	6.7 (5.4–8.3)	2.8 (2.1–3.6) **
White non-British	33.1 (22.0–44.2)	52.6 (36.5–68.7)	1.6 (1.1–2.2)	1.2 (0.9–1.5) †
Asian	31.6 (16.7–46.5)	54.5 (36.0–73.0)	1.5 (0.9–2.4)	1.4 (1.0–2.0)*
Mixed	45.9 (26.4–65.5)	52.1 (27.7–76.5)	2.7 (1.8–4.2)	1.4 (0.9–2.1) †
Other	55.0 (30.9–79.1)	126.4 (85.6–167.2)	2.6 (1.7–3.9)	4.1 (2.9–5.8)**

a ÆSOP was conducted from 1997 to 1999 (Fearon et al., 2006), the CRIS-FEP study was conducted from 2010 to 2012.

†*p* ⩽ 0.1; *p ⩽ 0.05; **p ⩽ 0.01.

These findings have changed to varying extents in the present CRIS-FEP study. The most striking was that rates for the white British group were higher (44.3 per 100 000 PY), while for the black Caribbean group (100.4 per 100 000 PY; adj. IRR 2.8; 95% CI 2.1–3.6) these were notably lower than those reported in the ÆSOP study. Also notable was the reduced magnitude of risk observed among the ‘mixed’ group from nearly three-fold (adj. IRR 2.7; 95% CI 1.8–4.2) to less than two-fold (adj. IRR = 1.4; 95% CI 0.9–2.1). While rates have increased for the Asian and white non-British groups, the rate ratios were broadly in keeping with the ÆSOP study. For the black African group, there was very little change in their RR from (adj. IRR 4.1; 95% CI 3.2–5.3) to (adj. IRR 3.59; 95% CI 2.8–4.5). By contrast, rate and rate ratios were considerably higher for the ‘other’ group (126.4 per 100 000; 95% CI 85.5–167.2) in the present study, but these need to be treated cautiously given the wide CIs.

Table 3 compares adjusted IRR in minority ethnic groups between the ÆSOP and CRIS-FEP studies, stratified by gender. In the ÆSOP study, raised IRR for black Caribbean, black African, ‘mixed’ and ‘other’ ethnic groups were present for both men and women. Whilst elevated rates by the ethnic group were also observed in the CRIS-FEP study, there were striking differences by gender when compared with the ÆSOP study. For example, there were notable reduction in the RRs for black Caribbean men: from nearly six-fold (adj. IRR 5.6; 95% CI 4.2–7.5) to less than threefold (adj. IRR 2.7; 95% CI 1.8–4.1), black Caribbean women: from eight-fold (adj. IRR 8.1; 95% CI 5.9–11.1) to less than threefold (adj. IRR 2.6; 95% CI 1.8–3.8) and black African women: from four-fold (adj. IRR 4.2; 95% CI 2.8–6.4) to less than threefold (adj. IRR 2.8; 95% CI 2.0–4.0). There was no change for black African men.

**Table 3.** Comparisons between ÆSOP and CRIS-FEP for adjusted IRR with 95% CI in ethnic minority groups for all psychoses, stratified by gender

	ÆSOP (*Fearon et al., 2006*)	CRIS-FEP study
Men		
White British	1.00	1.00
Black African	4.0 (2.9–5.7)	4.3 (3.1–6.0)**
Black Caribbean	5.6 (4.2–7.5)	2.7 (1.8–4.1)**
White non-British	1.7 (1.1–2.5)	1.3 (0.9–1.9)
Asian	1.3 (0.7–2.4)	1.2 (0.7–2.1)
Mixed	2.7 (1.6–4.7)	1.5 (0.9–2.7)
Other	2.4 (1.6–4.1)	5.9 (3.9–9.1)**
Women		
White British	1.00	1.00
Black African	4.2 (2.8–6.4)	2.8 (2.0–4.0)**
Black Caribbean	8.1 (5.9–11.1)	2.6 (1.8–3.8)**
White non-British	1.5 (0.8–2.5)	1.0 (0.6–1.5)
Asian	1.9 (0.9–3.7)	1.5 (0.9–2.5)†
Mixed	2.7 (1.4–5.4)	1.2 (0.6–2.5)
Other	2.9 (1.5–5.7)	2.0 (1.0–4.1)*

†*p* ⩽ 0.1; *p ⩽ 0.05; **p ⩽ 0.01.

### Age-specific IRR by ethnic group

Table 4 shows age-specific IRR by the ethnic group; we compared these to those reported in the ÆSOP study. Findings from ÆSOP showed that IRR were elevated for both black African and black Caribbean populations across all age groups. However, our findings from the CRIS-FEP study indicated that these were only elevated for the black Caribbean and black African groups in the age-bands 20–24, 25–29, 30–34, 35–39 and 40–44 years. Moreover, in the CRIS-FEP study, patients aged 25–29 and of Asian (adj. IRR 2.3; 95% CI 1.1–5.1) and mixed (adj. IRR 3.3; 95% CI 1.5–7.4) ethnic groups also had elevated rates. In addition, older (55–59 years) Asian patients (adj. IRR = 4.6; 95% CI 1.0–20.9) had higher rates, but no other ethnic minority group showed an increase in rate ratios in this particular age group.

**Table 4.** Age-specific and gender-adjusted IRR with 95% CI in ethnic minority groups for all psychoses: comparisons between ÆSOP and CRIS-FEP

Age band (years)	Black African	Black Caribbean	White non-British	Asian	Mixed	Other
ÆSOP (Fearon *et al.*, 2006)						
16–19	2.8 (1.2–6.3)	6.2 (3.6–10.9)	1.2 (0.3–4.9)	1.7 (0.6–4.7)	1.9 (0.7–5.4)	2.3 (0.7–7.5)
20–24	3.7 (2.0–6.9)	7.4 (4.6–11.8)	0.9 (0.4–2.2)	1.4 (0.6–3.5)	4.6 (2.3–8.9)	1.4 (0.5–3.9)
25–29	7.1 (4.3–7.4)	8.5 (5.1–14.1)	1.5 (0.7–3.0)	1.2 (0.4–4.0)	2.6 (0.9–7.3)	2.6 (0.9–7.2)
30–34	4.1 (2.3–7.4)	6.1 (3.6–10.4)	2.1 (1.1–4.2)	1.0 (0.3–4.1)	1.6 (0.4–6.4)	5.3 (2.3–12.5)
35–39	3.4 (1.5–7.4)	5.0 (2.7–9.4)	1.9 (0.8–5.0)	2.0 (0.5–8.3)	2.7 (0.6–11.2)	6.0 (2.1–17.1)
40–44	3.2 (1.2–8.5)	3.2 (1.3–7.9)	2.5 (0.9–7.4)	2.6 (0.6–11.3)	2.5 (0.3–18.4)	No cases
45–49	No cases	6.1 (2.2–17.1)	2.1 (0.4–2.4)	1.9 (0.3–14.3)	No cases	No cases
50–54	10.3 (2.1–8.5)	23.2 (7.7–69.4)	4.4 (0.9–21.3)	No cases	No cases	7.6 (0.9–61.6)
55–59	4.3 (0.5–33.8)	7.0 (1.9–25.9)	1.6 (0.2–12.5)	No cases	No cases	No cases
60–64	No cases	7.3 (2.1–24.8)	No cases	4.2 (0.5–34.3)	No cases	No cases
CRIS-FEP study						
18–19	0.79 (0.2–2.1)	0.64 (0.2–1.9)	0.79 (0.2–2.8)	0.38 (0.1–1.7)	0.49 (0.1–2.1)	No cases
20–24	4.37 (2.6–7.3)**	3.26 (1.8–5.8)**	1.06 (0.5–2.1)	1.25 (0.6–2.5)	1.45 (0.6–3.3)	4.98 (2.3–10.6)**
25–29	8.10 (4.5–14.3)**	4.64 (2.3–9.2)**	1.09 (0.5–2.2)	2.37 (1.1–5.1)*	3.36 (1.5–7.4)**	9.84 (4.7–20.2)**
30–34	4.83 (2.5–9.0)**	4.37 (2.1–8.9)**	1.11 (0.5–2.2)	1.94 (0.8–4.4)	0.41 (0.1−3.1)	2.77 (0.93–8.2)†
35–39	3.76 (1.9–7.3)**	3.55 (1.6–7.6)**	1.59 (0.7–3.3)	1.18 (0.4–3.5)	1.53 (0.4–5.3)	5.78 (2.4–13.6)**
40–44	4.24 (1.9–9.4)**	3.49 (1.4–8.4)**	2.86 (1.2–6.8)	3.06 (1.0–9.1)*	0.86 (0.1–6.8)	5.06 (1.5–16.4)**
45–49	1.84 (0.7–4.6)	1.55 (0.5–4.1)	1.40 (0.4–4.1)	0.68 (0.1–5.3)	1.70 (0.3–7.7)	2.58 (0.5–11.8)
50–54	0.86 (0.3−2.3)	1.17 (0.4–2.8)	1.01 (0.3–2.7)	0.44 (0.1–3.3)	1.40 (0.3–6.1)	2.07 (0.4–9.0)
55–59	2.10 (0.3–11.5)	2.82 (0.6–12.6)	1.92 (0.3–10.5)	4.67 (1.0–20.9)*	No cases	No cases
60–64	2.98 (0.5–16.3)	No cases	No cases	No cases	No cases	No cases

†*p* ⩽ 0.1; *p ⩽ 0.05; **p ⩽ 0.01.

## Discussion

### Main findings

We carried out a comprehensive study of the treated incidence of psychotic disorders using a large database of electronic health records of people who presented to secondary mental health services. Our results suggest that the overall incidence of psychotic disorders in southeast London has gone up between 1996–1999 and 2010–2012 and that rates have changed over time in all ethnic groups. The first, and perhaps most striking, findings were that rates were lower in the black Caribbean and higher in the white British populations; as a consequence, the rate ratios have narrowed. These changes were more marked by gender. That is, the magnitude of rate ratios particularly reduced for black Caribbean men and women and for black African women.

### Relationship with other previous studies

In keeping with some recent studies of incidence of psychosis in the UK, our results confirm that rates of psychosis are higher in black African and, to a lesser degree, black Caribbean populations, compared with white British population (Coid *et al*., 2008; Kirkbride *et al*., 2017). Regarding age and gender, our findings are consistent with previous studies (Coid *et al*., 2008; Cheng *et al*., 2011). We found that rates were at their peak among men between the ages 18–35 years, which mirrors previous findings (Fearon *et al*., 2006; Kirkbride *et al*., 2006; Coid *et al*., 2008). We observed elevated rates among women over the age of 39; the peak risk of psychosis for women has been reported to be close to the time of menopause (Grigoriadis and Seeman, 2002). Our findings also suggest that rate incidence of psychosis is not in declining, in keeping with some (Boydell *et al*., 2003; Kirkbride *et al*., 2009; Kirkbride *et al*., 2012), but not other, earlier studies (Geddes *et al*., 1993; Munk-Jorgensen and Mortensen, 1993).

### Methodological considerations

The primary methodological consideration is the comparability of methods of case ascertainment in AESOP and CRIS-FEP. It is possible that bias arising from differences in case ascertainment between the two studies (i.e. face-to-face *v.* case register) may explain the apparent changes in rates and rate ratios over time. Indeed, case register studies do tend to produce higher estimates of incidence. However, several points and lines of reasoning suggest this is unlikely to fully account for our findings. For example, it is the case register studies from Canada, the Netherlands and Scandinavian countries, that use linked national registers and rely on clinical diagnoses, that tend to produce higher estimates (Hogerzeil *et al*., 2014; Anderson *et al*., 2018; Jongsma *et al*., 2019); they do not allow for researchers to check case records to determine caseness and therefore may be over-inclusive. As noted earlier, in CRIS-FEP, we painstakingly reviewed records of every potential case to determine inclusion, with borderline cases being decided by consensus, thereby closely mirroring the approach used in ÆSOP. We also used identical inclusion and exclusion criteria in the two studies. Further, the changes in incidence we observed go in different directions for different ethnic groups (e.g. increase for white British, decrease for black Caribbean). It is not clear why the use of electronic records to identify cases, would over-include some and under-include other groups. While not discounting the possibility that using electronic records, rather than face to face screening of services, may explain some of the reported changes, we think it unlikely to be the sole explanation. Given what we know about the influence of environmental factors on psychoses and variations in rates across populations, it is reasonable to expect that rates will change over time. Our findings suggest that there are variations by the ethnic group in changes over time and, in doing so, challenge the usual framing of this issue, which implies elevated rates in minority ethnic groups are universal and static over time. There are other methodological considerations. For instance, our findings for the ‘Other’ ethnic group need to be considered with caution. It is a highly heterogeneous group and includes some newly added ethnic groups in the 2011 UK Census, e.g. Arab, and so it is possible that the denominator data may not be accurate; therefore, this finding may be artificially inflated if under enumeration is present in the denominator population. In addition, patients in our Asian group are heterogeneous, which in addition to Indian, Pakistani and Bangladeshi people also included those of Chinese origin.

A further potential limitation is in the lack of adjustment for socioeconomic variables, which may possibly explain differences in incidence rates by the ethnic group. However, where investigators have adjusted for socioeconomic factors in previous studies, this made little difference to the estimates (Kirkbride *et al*., 2008; Hollander *et al*., 2016; Kirkbride *et al*., 2017). Further, we deliberately based our case identification on broadly defined psychosis so as not to miss cases. Further research exploring rates of psychotic disorders by diagnosis is needed.

### Interpretations

It is possible that changes in incidence rates are related to changes in the demography of the source population, in mental health service provision, and in the distribution of environmental risk factors over time in the catchment area. The white British population in Lambeth and Southwark changed notably between the censuses of 2001 and 2011, when the proportion of White British people decreased from 50% to 39% (ONS, 2011*b*). This population change has potentially significant implications for changes in overall incidence rates (Kendell *et al*., 1993). Furthermore, the catchment area of southeast London contains some of the most socioeconomically deprived wards in England and Wales. Inevitably, many white British people are exposed to and may experience similar levels of socioeconomic disadvantages experienced by the minority ethnic groups (Social Mobility Commission, 2016), which may explain the higher rates in this group compared with 10 years ago. We demonstrated this in our earlier paper, where we found that the proportion of white British patients with first episode psychosis in southeast London who were unemployed rose from 53.7% to 63.9% between 1997–1999 and 2010–2012 (Oduola *et al*., 2019).

Further, UK government policy changes and major new investments in mental health services in the last two decades (Dept. of Health and Social Care, 1999) may have impacted on incidence rates of psychosis. For example, early intervention (EI) for psychosis services was established in southeast London in the early 2000s (Craig *et al*., 2004), the latter stage of the ÆSOP study. A central tenet of the EI services is early detection and reduction in delays to receiving treatment for people at an early stage in the psychotic illness. EI services are also known to work collaboratively with other agencies such as the criminal justice system and emergency rooms to identify people at the early phase of psychosis (Jarrett *et al*., 2012). Consequently, these services may identify more patients than was previously possible, which may explain the overall higher incidence rates. However, this is unlikely to fully explain the observed changes. As noted above, changes in rates varied by the ethnic group; therefore, to fully explain, for example, increased rates in the white British group and decreased rates in the black Caribbean, EI services would need to be more engaging for white people; they would also have to be less engaging than mainstream services for black Caribbean (but not black African).

Another possibility is that this increase is linked to the rise in the use of high-potency cannabis, which has been linked to the onset of psychosis (Marconi *et al*., 2016; Murray *et al*., 2017). In particular, a recent case-control study of incidence of psychosis in south London, showed that cases who used skunk-like cannabis daily had up to five times increased odds of psychotic disorder compared with those who never used cannabis (Di Forti *et al*., 2015). Around the same time of our study, the samples of cannabis seised by the Metropolitan Police in London area had higher concentration of tetrahydrocannabinol than those in the late 1990s when AESOP was carried out (Freeman *et al*., 2014; Di Forti *et al*., 2015).

The reduced incidence of psychosis in the black Caribbean population that we observed may be explained by considering generational status. Although not measured specifically here, the majority of the black Caribbean group in our sample and in the UK population are second or third generation, since migration among black Caribbean populations into the UK was highest post World War II, mostly in the 1950s (Chamberlain, 2002; Coid *et al*., 2008; Jones, 2011). Therefore, it is possible that social networks for the black Caribbean group may have improved, which has been documented to be a protective factor from mental illness (Bhugra and Becker, 2005). We showed in our recent study that black Caribbean patients are now more likely to live with family and friends (41.7% *v.* 61.1%), compared with 15 years ago (Oduola *et al*., 2019). In addition, the lower IRR among the black Caribbean group are partly explained by the higher incidence rate that we observed among the white British group in this study compared with the ÆSOP study. Since they are the reference group, a rise or drop in the incidence rate of psychosis for the white British groups will affect the IRR in the other ethnic groups.

It is also possible that other sociodemographic changes in London may have influence rates of psychosis in some ethnic groups. According to the Indices of Deprivation (2015), London has relatively low levels of deprivation in education (Ministry of Housing and Local Goverment, 2015). Data from our recent study showed that the proportion of first episode psychosis patients with university level of education rose from 14.9% in AESOP to 27.5% in CRIS-FEP (Oduola *et al*., 2019), which may suggest an improvement in this well documented social risk factor of psychosis. However, this may at most only partly explain our findings, since elevated rates are still present in some minority ethnic groups but not others, as discussed above.

Furthermore, the fact that we observed little or no change in the overall magnitude of risk of psychosis among black African patients may be explained in a number of ways. First, for the black African patients, it is possible that the well documented indicators of social disadvantage (Morgan *et al*., 2009), isolation (Reininghaus *et al*., 2008) and discrimination (Reininghaus *et al*., 2010) may be driving the excess of psychosis observed among the black African and other minority ethnic populations in our study. According to the Office for National Statistics (2005), there has been an increase of 2.4% in the black African population in the catchment area since the 2001 census (ONS, 2011*b*), indicating this is an active migrant group. It has also been reported that recent migrants may be predisposed to such experiences on arrival in the new environment since there may be tension with regard to cultural bereavement and culture shock i.e. a discrepancy between expectations and achievement in the host country (Bhugra and Becker, 2005). A recent study from Sweden illuminated this issue, where the authors investigated the rates of schizophrenia and non-affective psychosis in refugee and non-refugee migrants compared with native Swedish populations. They found that refugee migrants had higher rates of psychosis than non-refugee migrants, but the risk was particularly greater for those from sub-Saharan Africa irrespective of refugee status (Hollander *et al*., 2016). Despite not controlling for country of birth or generation status in this study, our findings here for the black African patients are consistent with previous studies.

Despite the increase in the white non-British population in the UK, particularly since the expansion of the European Union in 2004 and ease of migration from Eastern Europe, we found weak evidence of increased risk of psychosis between the white non-British and white British ethnic groups. Our findings in this group are in keeping with two recent studies (Kirkbride *et al*., 2017; Schofield *et al*., 2017), which both found that non-British white ethnic groups in the UK and migrants from elsewhere in Europe to Denmark were not at increased risk of psychosis. This may also be because the white non-British patients may experience a less stressful acculturation process and lower perceived discrimination (Schofield *et al*., 2017) within Europe.

## Conclusions

Our results suggest that incidence rates of psychosis are still elevated among minority ethnic groups. However, the magnitude of IRR varies considerably by the ethnic group. The findings here also highlight that black African and other ethnic groups remain disproportionately at higher risk of psychosis compared with their white British counterparts. Our findings suggest that concerted efforts are needed to ameliorate health outcomes for our minority populations, and could also help inform commissioners, policymakers and healthcare providers in allocating resources to delivery effective mental health services and public health strategies. While our study has shed light on possible demographic changes over time to explain change in incidence of psychotic disorders, future studies are needed to investigate more changes over time and determinants of change.

## Supplementary material

For supplementary material accompanying this paper visit https://doi.org/10.1017/S0033291719003234. click here to view supplementary material
