# Supplementary material for: Change in incidence rates for psychosis in different ethnic groups in south London: findings from the Clinical Record Interactive Search-First Episode Psychosis (CRIS-FEP) study
Source: Psychol Med. 2019 Nov 19;51(2):300–9. doi: 10.1017/S0033291719003234 (PMC7893508; doi:10.1017/S0033291719003234)
Supplement: Supplementary file 1 [file S0033291719003234sup001.docx]

Supplementary Figure 1: Screening flowchart: CRIS-FEP study

**Screened positive** (n=560)

**Patients eligible for screening** (n=9109)

**Final FEP cases** (n=558)

**Excluded (n=2)**

Insufficient information (n=2)

**Excluded (n= 8549)**

No psychosis (n=5234)

Previous psychosis, (n=2556)

Not resident, aged <18 or >64 (359)
